# Promotion of Preventive Sleep Research from the Perspective of Health and Public Safety in Japan - Secondary Publication

**DOI:** 10.31662/jmaj.2024-0200

**Published:** 2025-07-09

**Authors:** Takeshi Tanigawa

**Affiliations:** 1 Department of Public Health, Juntendo University Graduate School of Medicine, Tokyo, Japan

**Keywords:** sleep-disordered breathing, traffic accident, lifestyle diseases, screening test

## Abstract

Ensuring good sleep quality and adequate sleep duration is crucial for health. Sleep apnea syndrome (SAS) impairs sleep quality and increases the risk of diabetes, cardiovascular diseases, and accidents. The author has significantly advanced the understanding of SAS in Japan through over 20 years of epidemiological studies. Findings have revealed that individuals with a 3% oxygen desaturation index (ODI) ≥15 events/h have a 1.69-fold higher risk of developing diabetes. Those with mild to severe sleep-disordered breathing (SDB) with a 3% ODI ≥5 events/h face high risks of heart disease and lacunar infarction, at 26.1% and 30.1%, respectively. Among shift workers aged ≥40, SDB was significantly associated with elevated blood pressure. Additionally, the risk of traffic accidents in SDB patients is approximately 2.5 times higher than in those without SDB. The author advocated for routine SAS screening, especially for professional drivers, to enhance public safety. In collaboration with the Ministry of Land, Infrastructure, Transport and Tourism, comprehensive guidelines for SAS screening were developed, emphasizing the necessity of objective methods due to the disconnect between subjective sleepiness and objective alertness. These methods include pulse oximetry and the 3-minute psychomotor vigilance test (PVT). Highlighting the health risks for night-shift workers with SDB, the author promoted regular screening and early intervention. Additionally, pediatric SAS was linked to attention-deficit hyperactivity disorder-like symptoms, stressing the need for early treatment. Post-disaster health initiatives showed increased insomnia among Fukushima recovery workers, with continuous positive airway pressure therapy proving beneficial. The author also developed health security measures using PVT for doctors working long hours, linking reduced wakefulness to depression and burnout. These contributions have significantly improved public health and safety in Japan, influencing health policies and promoting widespread SAS screening and sleep debt evaluation. Continued support from doctors of the Japan Medical Association is essential to maintain these advancements.

## Introduction

To ensure adequate sleep, it is necessary to secure both good sleep quality and sufficient sleep duration. Sleep apnea syndrome (SAS) is an important pathological condition that reduces sleep quality. In addition, SAS has been shown to be a risk factor for traffic and industrial accidents, as well as cardiovascular and lifestyle-related diseases. From the viewpoint of public safety and overall health, SAS is an issue that should be addressed not only for individual health but also to ensure safety and security for society as a whole.

## Sleep-disordered Breathing (SDB) and SAS

SDB is a general term used to describe abnormal respiratory conditions during sleep. SAS is diagnosed when symptoms such as daytime sleepiness and fatigue are present in addition to SDB. Recently, the term SDB has been changed to “sleep-related breathing disorder” according to the International Classification of Sleep Disorders. However, the term SDB is used in this article. While the number of patients with SAS in Japan is estimated to be between 4 million and 5 million, the number of patients currently receiving treatment is only around 0.6-1 million. Furthermore, as SDB gradually worsens, the resulting sleepiness is likely to be mistaken for age-related chronic fatigue. In addition, patients are often unaware of subjective sleepiness, and SDB is likely to be overlooked in medical interviews alone ^(1)^. Therefore, it is necessary to establish a system for routine SDB screening tests to prevent traffic and industrial accidents as well as lifestyle-related diseases, even in the absence of subjective sleepiness.

## SDB and Health

Recent epidemiological studies have shown that SDB is a risk factor for hypertension, glucose intolerance, arteriosclerosis, atrial fibrillation, ischemic heart disease, and stroke. The author initiated epidemiological studies in 2000, ahead of other countries, monitoring arterial oxygen saturation during sleep using pulse oximetry. The number of times per hour that oxygen saturation drops transiently (oxygen desaturation index [ODI]) was used as an indicator of SDB severity. The author has published approximately 60 original articles on sleep-related issues, and the major articles are listed in the Table 1. In addition, the “Sleep Apnea Syndrome Screening Handbook” ^(2)^ was published to encourage the implementation of SAS screening at health checkup facilities and other medical institutions nationwide.

**Table 1.** Major Publications.

Themes	Journal title	Year of publication	Volume (number): page.
** Risk factors for Sleep-disordered breathing**			
Alcohol consumption and sleep-disordered breathing	JAMA	2004	292(8):923-925.
Facial skeleton, BMI, and sleep-disordered breathing	Laryngoscope	2004	114(10):1838-1842.
Alcohol consumption and sleep-disordered breathing in truck drivers	Alcohol Clin Exp Res	2007	31(12):2053-2058.
Weight change since age 20 and sleep-disordered breathing in truck drivers	Int J Obes (Lond)	2009	33(12):1396-1401.
Comparison of the prevalence of sleep-disordered breathing in Japan and the US	Eur Respir J	2010	36(2): 379-384.
Alcohol consumption and sleep-disordered breathing in women	Respir Med	2011	105(5):796-800.
Teeth mark tongue and nocturnal intermittent hypoxia	J Oral Rehabil	2017	44(8): 602-609.
** Sleep-disordered breathing and cardiovascular risk factors**			
Sleep-disordered breathing and blood pressure	Hypertens Res	2004	27(7): 479-484.
Sleep-disordered breathing and blood pressure/excessive sleepiness in truck drivers	Hypertens Res	2006	29(8): 605-610.
Sleep-disordered breathing and blood pressure in shift/day workers	Am J Hypertens	2006	19(4): 346-351.
Sleep-disordered breathing and atrial fibrillation	Heart	2006	92(12):1854-1855.
Sleep-disordered breathing and high-sensitivity CRP	Sleep	2006	29(5): 661-665.
Sleep-disordered breathing and blood pressure/excessive sleepiness in women	Hypertens Res	2008	31(3): 501-506.
Nocturnal intermittent hypoxia and onset of type II diabetes	Diabetologia	2010	53(3): 481-488.
Nocturnal intermittent hypoxia and metabolic syndrome; Influence of obesity	J Atheroscler Thromb	2010	17(4): 369-377.
Nocturnal intermittent hypoxia and microalbuminuria in diabetic patients	Eur J Endocrinol	2013	169(2): 239-246.
Sleep-disordered breathing and ANP	Int J Cardiol	2014	173(2): 334-335.
Nocturnal hypoxia and arteriosclerosis	J Atheroscler Thromb	2014	21(12):1290-1297.
Nocturnal intermittent hypoxia and impaired glucose tolerance	Sleep Med	2014	15(10):1212-1218.
Association between sleep-disordered breathing and central blood pressure	Hypertens Res	2019	42(7): 1074-1082.
Effects of sleep-disordered breathing and drinking on blood pressure	Hypertens Res	2021	44(9): 1168-1174.
CPAP treatment and blood pressure in SAS patients	Sci Rep	2021	11(1): 1531.
Sleep-disordered breathing and risk of cardiovascular disease	J Atheroscler Thromb	2022	30(9): 1276-1287.
** Sleep-disordered breathing and traffic accidents**			
Nocturnal intermittent hypoxia and psychomotor vigilance	Sleep Med	2017	29: 7-12.
Association between sleep-disordered breathing and traffic accidents	Sci Rep	2020	10(1): 17050.
Poor prediction of self-reported sleep tendency for obstructive sleep apnea in truck drivers	Sleep Med	2024	115:109-113.
** Sleep-disordered breathing and behavioral problems among children**			
Sleep Duration, Snoring Prevalence, Obesity, and Behavioral Problems in a Large Cohort of Primary School Students in Japan.	Sleep	2017	40(3): zsw082.
Association between nocturnal enuresis and sleep-disordered breathing in children	Pediatr Pulmonol	2018	53(11):1541-1548.
Sleep-disordered breathing and upper respiratory infection in children	Sleep Breath	2024	28(2):629-637.
** Others**			
Automatic determination of sleep-disordered breathing with a 1-channel breathing sensor	Eur Respir J	2007	29(4): 728-736.
Appropriateness of screening for sleep-disordered breathing with a 1-channel breathing sensor	Eur Respir J	2008	32(4): 1060-1067.
Prevalence of sleep-disordered breathing in nursing home female staff	Int Arch Occup Environ Health	2019	92(3): 309-316.
Association between pectus excavatum and sleep-disordered breathing in children	Eur Respir J	2019	54(4): 1900524.
PVT and sleep-disordered breathing in nursing care staff	Sleep Breath	2022	26(1): 259-267.
Improvement of insomnia by CPAP at evacuation centers during a disaster	Sleep Health	2023	S2352-7218(23)00124-9.

### SDB and diabetes

The author and colleagues performed pulse oximetry on approximately 4,000 local residents and followed them for approximately 4 years. The relative risk of developing new-onset of diabetes mellitus in the group of people with a 3% ODI ≥15 events/h was 1.69, even after adjusting for potential risk factors ^(3)^. The population-attributable risk percentage of diabetes in the group with a 3% ODI ≥5 events/h was 11.5%. This indicates that early detection and treatment of mild-to-severe SDB may prevent the onset of diabetes mellitus by approximately 10% in Japan.

### SDB and cardiovascular diseases

The results of the Circulatory Risk in Communities Study, a community epidemiological study started in the 1960s by the late Yoshio Komachi, professor emeritus at the University of Tsukuba, which followed approximately 5,000 local residents for 20 years, showed that people with mild-to-severe SDB (3% ODI ≥5 events/h) had a high population-attributable risk of heart disease and lacunar infarction, at 26.1% and 30.1%, respectively. They also found that the effect of mild-to-severe SDB on the onset of cardiovascular disease was comparable to that of hypertension and smoking ^(4)^. Currently, in Japan, the apnea-hypopnea index (AHI) of ≥ 20 events/h, as determined by polysomnography, is the criterion for health insurance coverage of continuous positive airway pressure (CPAP) therapy. However, since the population-attributable risk percentage of heart disease and lacunar infarction is high when 3% ODI ≥5 events/h, which is almost comparable to AHI ≥5 events/h, reasonable criteria for health insurance coverage of CPAP should be discussed in the future to prevent cardiovascular diseases.

### SDB and night-shift work

In Japan, approximately 20% of employees work late at night on a rotational basis. Night-shift work is required in a wide range of fields, including transportation, airlines, medical care, nuclear power plants, manufacturing, and security. However, safety and health measures for night-shift work remain major concerns, as it is a risk factor for cardiovascular diseases and can cause or exacerbate hypertension in workers with SDB. In our survey, SDB was significantly associated with increased blood pressure in shift workers aged ≥40 years ^(5)^. For occupations that involve night-shift work, it is appropriate that workers undergo SDB screening, leading to early detection and treatment, regardless of the presence or absence of subjective symptoms ^(6)^.

### SDB and traffic accidents

The risk of traffic accidents is approximately 2.5 times higher in patients with SDB ^(7)^. However, accidents directly attributed to SDB represent only a small portion of all SDB-related accidents. Many accidents caused by SDB have been handled without recognizing the relationship to SDB, making it difficult to prevent recurrence.

SAS screening conventionally uses the Epworth Sleepiness Scale (ESS), which assesses subjective sleepiness. After an incident of drowsy driving involving a Shinkansen bullet train driver in 2003, more workplaces began using the ESS. However, in a train collision in Gifu caused by drowsy driving in October of the same year, the driver was assessed as having no pathological sleepiness by the ESS prior to the incident and was exempt from SAS screening. Nevertheless, the driver was later diagnosed with severe SAS based on a detailed examination after the accident.

When a questionnaire was conducted among patients receiving SAS treatment regarding traffic accidents and near misses, it was revealed that many of them fell asleep unknowingly. Some reported that by the time they became aware, they had collided with a car at a red light in front of them. When interviews to determine subjective sleepiness (ESS) and a brief objective SAS screening test using the flow sensor method were simultaneously conducted among approximately 5,000 truck drivers, 76% of drivers rated their subjective sleepiness as normal despite having severe SDB (respiratory event index ≥40 events/h) ^(1)^. It has also been reported that subjective sleepiness is dissociated from objective alertness in cases of long-term sleep deficiency ^(8)^. Thus, SAS screening tests using questionnaires to assess subjective sleepiness are not effective, and objective screening tests using pulse oximetry or flow sensors are required. Pulse oximetry often underestimates the severity of SDB in non-obese individuals because oxygen desaturation is less pronounced than in those who are obese. In contrast, flow sensor testing monitors airflow during sleep using sensors attached to the nose and mouth to determine the degree of apnea and hypopnea. The detection accuracy of the flow method is particularly high in non-obese individuals ^(9), (10)^. The author initiated the world’s first SAS screening test for truck drivers using the flow sensor method in 2002. Due in part to the author’s efforts ^(2)^, SAS screening is now performed in many health examination centers. Currently, in Japan, more than 100,000 professional drivers undergo SAS screening annually, facilitating the early detection and treatment of SDB.

## Efforts against SAS in Japan

At the request of the Ministry of Land, Infrastructure, Transport and Tourism (MLIT), the author prepared a manual titled “Let’s watch for sleep apnea syndrome (SAS)” immediately after a Shinkansen driver was reported to have experienced drowsy driving on February 26, 2003. The author’s efforts helped disseminate the importance of preventing SAS-related accidents within the transportation industry. Moreover, after discovering that many individuals with SDB do not subjectively perceive sleepiness, the author revised the manual in 2007 to raise awareness of unpredictable sleepiness, and emphasize the importance of objective screening methods ^(11)^. Subsequently, the MLIT established the Review Committee on Measures Against Health-Related Accidents to promote measures against accidents caused by health conditions, including SAS. The author of this paper also participated as a member of this committee. In 2015, the MLIT released the “Manual for Measures against Sleep Apnea Syndrome in Automobile Transportation Operators: Necessity and Utilization of SAS Measures” ^(12)^, which provides a detailed framework for SAS screening, from pre-screening to post-screening actions. The MLIT has continued to improve driver safety and health by expanding SAS screening and encouraging appropriate treatment. The author highlighted the importance of SAS measures in the Interim Assessment Report on “Healthy Japan 21 (2nd term)” ^(13)^. However, SAS screening has not yet been fully integrated into the Ministry of Health, Labour and Welfare’s occupational health initiatives. Patients with mild-to-moderate SDB have largely remained untreated. Given the high prevalence of SDB and its strong association with cardiovascular diseases, early detection and treatment should be prioritized, even for mild cases.

For health examinations mandated by employers under the Industrial Safety and Health Act, a benefit system allows employees to receive free-of-cost secondary health examinations and specific health guidance if they are diagnosed with abnormal blood pressure, blood glucose, and blood triglyceride levels, all of which are related to the onset of stroke and coronary heart disease. These benefits, provided under the Workers’ Accident Compensation Insurance, aim to prevent brain and heart diseases caused by metabolic syndrome. However, it is considered essential to include SAS screening in secondary health examination items to reduce the risks of traffic and industrial accidents, as well as stroke and coronary heart diseases. Various institutions and organizations related to occupational health and administration are expected to promote early detection and treatment for SAS in the future.

## Industrial Health Activities at the Fukushima Daiichi and Daini Nuclear Power Plants of the Tokyo Electric Power Company after the Great East Japan Earthquake

In April 2011, the author began engaging in industrial health activities to relieve the stress of workers at the Fukushima Daiichi and Daini Nuclear Power Plants, who were involved in the recovery of the Tokyo Electric Power Company’s Fukushima Daiichi Nuclear Power Plant after the Great East Japan Earthquake in March of that year ^(14)^. It was found that the most stressful factor for employees working in recovery efforts during such an unprecedented major disaster was discrimination or verbal abuse from local residents ^(15)^. Additionally, the percentage of workers experiencing insomnia symptoms, which persisted for several years after the earthquake, was found to have increased ^(16)^. Furthermore, studies have shown that in disaster situations where people are forced to sleep in gymnasiums, the use of CPAP by individuals who snore loudly is effective in reducing insomnia among those sleeping nearby ^(17)^.

## Importance of Measures against Pediatric Sleep Health

During discussions with otorhinolaryngologists, the author and colleagues learned that pediatric SAS can cause attention-deficit hyperactivity disorder-like symptoms, prompting further investigation into this issue. An epidemiological study of approximately 20,000 children was conducted with the cooperation of the Education Committee, school principals in Matsuyama City, Ehime Prefecture, and the Matsuyama Otorhinolaryngological Association, along with the participation of parents from all elementary schools in the city. Based on medical interviews, the results showed that short sleep durations, obesity, and suspected SDB were significantly associated with problematic behaviors, such as restlessness and poor concentration ^(18)^. The author is currently investigating the relationship between objective wakefulness and mental and physical health in approximately 2,000 elementary school students in Koto-ku, a ward in the Tokyo Metropolis.

## Work Style Reform for Doctors: Health Assurance Measures for Doctors Working Long Hours

The author served as the chairperson of the preparation committee for the “Manual on Health Security Measures for Doctors Who Work Long Hours,” issued in December 2019, and the revised version ^(19)^, published in October 2023. This manual proposes utilizing the 3-minute psychomotor vigilance test (PVT) to objectively evaluate the sleep debt of doctors who work long hours and are subjected to insufficient sleep. In a study involving approximately 1,200 doctors nationwide, a decrease in objective wakefulness―based on 3-minute PVT results―was significantly associated with depression and burnout; however, no such association was found with reduced sleep duration ^(20)^. In the future, disseminating and promoting methods for objectively evaluating sleep debt will be essential to ensuring public health and safety.

## Conclusion

The author believes that promoting SAS screening for both adults and children, as well as implementing objective sleep debt screening at medical institutions and health checkup facilities across Japan, will contribute to improving public safety and health. Further support from the doctors of the Japan Medical Association is essential to advancing efforts in sleep health and safety.

## Article Information

This article is based on the study, which received the Medical Award of The Japan Medical Association in 2023. This is a revised English version of the article originally published in Japanese in the Journal of the Japan Medical Association 2024; 152(10):1155-9 ^(21)^. The original version is available at https://med.or.jp/cme/jjma/newmag/pdf/152101155.pdf. Only members of the Japan Medical Association are able to access it.

### Conflicts of Interest

None

### Acknowledgement

The author would like to express his sincere gratitude to the following doctors: the CEO, Hideoki Ogawa, for his warm support of research activities at Juntendo University, as well as other doctors who provided guidance and support during the author’s time as a student at Kobe University School of Medicine and the Graduate School of Medicine, University of Tokyo, and throughout his tenure at the University of Tsukuba and Ehime University. In addition, the author would like to express his deep appreciation for the cooperation of researchers who have contributed to previous joint studies, as well as the residents and workers in community and workplace settings who have participated in epidemiological research.
